# Characterising Potential Subtypes and Influencing Factors of Sleep Quality in Psychiatric Nurses by Latent Profile Analysis

**DOI:** 10.1155/2024/3842592

**Published:** 2024-07-18

**Authors:** Xiaoshi Pan, Jiayi Wang, Ke Zhang, Chenxin Yang, Minghong Tang, Zhaoxin Feng, Li Liu, Hui Wu

**Affiliations:** Department of Social Medicine School of Health Management China Medical University, Shenyang 110122, Liaoning, China

## Abstract

**Background:**

Sleep is a crucial factor affecting an individual's physical and mental health. Psychiatric nurses work under high stress and load, and it is necessary to understand the sleep quality of psychiatric nurses and the influencing factors. However, individual-centred studies of psychiatric nurses' sleep are limited.

**Aims:**

To explore the heterogeneity in sleep quality among psychiatric nurses, to identify the factors influencing different subtypes, and to provide targeted strategies and measures to improve their sleep quality.

**Methods:**

From August to October 2022, 298 psychiatric nurses working in a mental health centre in Liaoning Province were selected as the participants. The study involved administering the following two questionnaires: the general information questionnaire and the Pittsburgh Sleep Quality Index (PSQI). Data analyses included latent profile analysis, Kruskal–Wallis *H* test, and multiple logistic regression.

**Results:**

The prevalence of poor sleep quality (PSQI >5) among psychiatric nurses was 54.7%. The sleep quality of psychiatric nurses could be classified into the following three distinct profiles: good sleep quality, moderate sleep quality, and poor sleep quality. Nurses who were over 40 years of age, unmarried/divorced/separated/widowed, worked more than 40 hours per week, experienced significant life events in the past year, had poor nurse-patient relationships, and had chronic diseases were more likely to have poorer sleep quality.

**Conclusions:**

There was significant heterogeneity in sleep quality among psychiatric nurses. Age, marital status, work schedule, total weekly working hours, night shifts, special life events, nurse-patient relationships, and chronic diseases were associated with their sleep quality. *Implications*. The heterogeneity and influencing factors of sleep quality in psychiatric nurses provided evidence for individualized interventions in the future. This trial is registered with ChiCTR2200062347.

## 1. Introduction

Sleep as a periodic and reversible resting phenomenon is one of the most important life activities in human daily life. Chronic sleep problems accelerate aging and fat accumulation [1] and increase the occurrences of serious diseases of cardiovascular and cerebrovascular diseases [2, 3]. At present, scholars generally measure sleep quality at the subjective and objective levels [4]. However, objective definitions are hard to be used, as they are limited to physiological and behavioral aspects and are difficult to be obtained [5]. Thus, self-reported subjective feelings remain the most consistent measure of sleep quality [6]. On a subjective level, Buysse's definition has been the most widely used [7], which claims that sleep quality is an overall concept that integrates the seven components of subjective sleep quality, and assesses the level of sleep quality by the Pittsburgh Sleep Quality Index (PSQI). The cutoff scores of PSQI cutoff vary across populations [8], in which sleep quality of nursing staff PSQI >5 is the most commonly recognized [9].

Psychiatric nurses, who belong to a highly specialized occupational group, are required to care for patients with psychiatric problems and often behave abnormally with self-harm and aggressive behavior. In addition, some patients may also be accompanied by physical illness and complications. Psychiatric nurses need to attend to the various needs of patients and changes of patients' physical and mental states [10]. Encountered with numerous challenges, psychiatric nurses are subject to high tension and sleep deficit due to long hours of pressurized work [11]. According to studies, the prevalence of poor sleep quality among psychiatric nurses in China is 53.08%–71.50% [12, 13], significantly higher than that of general nurses in previous studies, which indicates that psychiatric nurses have serious sleep problems due to their professional particularity.

Poor sleep quality among psychiatric nurses damages their physical and mental health and affects the efficiency and safety of their nursing work [14]. The harmful impacts include the fluctuation of immune biomarkers [15], periodontal disease [16], headache [17], anxiety or depression [18], and cognitive impairment [19]. In addition, it can negatively affect nurses' attention, memory, judgment, and the ability to communicate effectively. As a result, they may become slower to respond, less motivated, less productive at work, and changes in the patient's condition may go unnoticed or even risk medical errors [20]. Therefore, improving the sleep quality of nurses and managing their occupational health has become a public health concern to the whole society.

Spielman [21] proposed the “3P” theoretical model of insomnia, indicating that individual sleep quality is affected by predisposing factors, precipitating factors, and perpetuating factors, and chronic insomnia will occur when these three types of factors interact [22]. Among them, the predisposing factors of insomnia refer to the personal characteristics of insomnia, that is, who is more prone to insomnia. The precipitating factors of insomnia refer to the causes of insomnia in the first place. The perpetuating factors are the factors that make insomnia last for a long time, including unhealthy behavioral habits and incorrect cognition. Based on the abovementioned theoretical model, we selected age, gender, marital status, education, and monthly income as the predisposing factors of poor sleep quality of psychiatric nurses; working age and working mechanism. Working hours per week, night shifts, special events, nurse-patient relationships and chronic diseases were cited as the precipitating factors [23, 24]. The abovementioned influencing factors on the sleep quality of nurses are the basic support for managers to intervene nurses' sleep.

At present, many studies on the sleep quality of nurses determine their sleep quality status based on the total score of sleep-related scales, and the exploration of the influencing factors of sleep quality of nurses also ignores the heterogeneity of their sleep-related problems [25, 26]. “Heterogeneity” means that individuals differ in their characteristics and attributes, leading to differences in behaviors, beliefs, needs, abilities, and other aspects. The existence of heterogeneity indicates that each individual may have different sleep quality problems [27].

Some studies have suggested an individual-centred approach rather than a variable-centred approach to differentiate between individuals in terms of heterogeneity in sleep quality. Some scholars have found group heterogeneity in the sleep quality of nurses; Han et al. [28] divided 465 nurses into a high-symptom group and a low-symptom group based on their scores on the General Sleep Disorder Scale. Slavish et al. [29] classified nurses' sleep status as “poor overall sleep,” “nightmares only,” “good overall sleep,” “only nightmares,” and “overall good sleep” based on their sleep diary records.

Although previous studies have provided us with information on the validity of the latent profile analysis (LPA), a person-centred method that reveals heterogeneous subgroups of individuals with the expressions of multifaceted features, the subtypes of sleep quality in psychiatric nurses are currently unknown. Due to the specificity of the patients they serve, psychiatric nurses are under higher levels of stress and their sleep quality is expected to be different from general nurses in other departments. Therefore, the identification of psychiatric nurses' sleep quality subtypes is important for the subsequent development of specific sleep interventions.

## 2. Materials and Methods

### 2.1. Design

This study is an exploratory, quantitative, individual-centred cross-sectional study.

### 2.2. Participants

The sample size was calculated by the following formula:

(1) $$\begin{array}{c} n=\frac{Z_{1-\alpha /2}^{2}\times p\left(1-p\right)}{d^{2}}. \end{array}$$

The confidence level was 95% (two-sided), *Z*_1−*α*/2_ (standard normal variate) was 1.96, *p* was 60% for the estimated prevalence of poor sleep quality among psychiatric nurses according to the results of previous studies, and *d* was 6% for the margin of error. Thus, we identified 257 as the minimum sample size for this study.

In addition, a 20% nonresponse rate was considered. In this study, 330 questionnaires were distributed to professional nurses from a mental health centre in Liaoning Province from August to October 2022. The inclusion criteria were as follows: (a) obtaining a nurse practitioner's license and having it in force; (b) nurses registered and working for ≥1 year; and (c) frontline nurses working in psychiatric wards. Exclusion criteria for nurses were as follows: (a) nurses on maternity leave, sick leave, sabbatical leave, study, and further training during the survey period and (b) trainee nurses. The researcher contacted hospital administrators to distribute the questionnaire to nurses who met the criteria. Nurses whose responses did not fit the internal logic of the questionnaire were excluded, and 298 valid questionnaires were actually collected with an effective rate of 90.3% in this study.

### 2.3. Instrument

#### 2.3.1. Sleep Quality

Sleep quality was measured using the Pittsburgh Sleep Quality Index developed by Busysse et al. [7], the Chinese version was revised and compiled in China by Liu and Tang, and the Cronbach's alpha coefficient of the seven main components and each item of the Chinese version of PSQI is higher than that of Buysse's test results and has high internal consistency, reliability, and validity. It is widely used in the Chinese population [30]. The PSQI comprises the following seven components: (A) subjective sleep quality (number of items = 1), (B) sleep latency (number of items = 2), (C) sleep duration (number of items = 1), (D) sleep efficiency (number of items = 2), (E) sleep medication (number of items = 9), (F) sleep disturbance (number of items = 1), and (G) daytime dysfunction (number of items = 2). Scoring involved 7 components, 18 items with each component receiving a score of 0–3 after secondary calculation. The total score ranged from 0 to 21, where higher scores indicated poorer sleep quality for individuals. Previous studies have established various cutoff values for the PSQI across different populations, ranging from 4 to 10 points. In the nursing population, it is generally accepted that a PSQI score >5 indicates poor sleep quality [31, 32]. The scale has been widely used to evaluate the sleep quality of nurses and has good reliability and validity. The Cronbach's alpha coefficient in the study was 0.876, so there was good internal consistency of the Chinese version of PSQI [30].

#### 2.3.2. General Information Questionnaire

The study utilized a self-designed general information questionnaire that focused on gathering demographic and sociological information from psychiatric nurses. The questionnaire included age (≤40 years and >40 years), length of service (≤10 years and >10 years), gender (male and female), marital status (married or cohabiting and unmarried/divorced/separated/widowed), education (junior college and below and bachelor's degree and above), monthly income (CNY) (≤5000 yuan, >5000 yuan), work schedule (fixed schedule and shift work), total weekly working hours (≤40 h and >40 h), night shifts (yes and no), whether experienced any special life events such as the death, illness of a relative, promotion, and marriage of children within the past year (yes and no), nurse-patient relationship (good and poor), and whether they suffered from chronic diseases such as hypertension, diabetes, and arthritis (yes and no).

### 2.4. Data Analysis

This study used SPSS 21.0 and Mplus 8.3 for data analysis. The enumeration data were described as *n* (%), and the normality of measurement data was tested by Kolmogorov–Smirnov. The normal data were expressed as mean and standard deviation (SD), and the skewed data were expressed as the median and interquartile range. We first conducted the normality test and common method bias test on the data and conducted latent profile analysis with the scores of each dimension of sleep quality of psychiatric nurses as the dominant variables. LPA is a statistical method that is based on multiple continuous indicators to assess individual characteristics across different components. Through probabilistic calculations, it identifies the subgroup to which an individual is most likely to belong. This method aims to classify individuals into distinct subtypes by identifying similarities in their characteristics. The best classification model is selected based on the merits of the fit indices and the practical significance of the profile.

Table 1 shows the model fit indices [33, 34]. The sleep quality of psychiatric nurses in the three groups did not conform to the normal distribution (*P* < 0.05), so the median and interquartile range were used to describe the sleep quality of nurses. Then, the Kruskal–Wallis *H* test was used for one-way analysis of variance, and the Dunn's method with a Bonferroni correction for multiple tests was used for post hoc test to compare the differences in demographic characteristics among different subtypes; the statistically significant variables were included in the multivariate multiple logistic regression analysis to identify the influencing factors between different sleep quality subtypes. Two-tailed *P* < 0.05 was considered statistically significant.

### 2.5. Ethical Considerations

Ethical approval for this study was obtained from the Research Ethics Committee of China Medical University. Informed consent was completed by all participants. The registered number is ChiCTR2200062347.

## 3. Results

### 3.1. Common Method Bias Test

The data were tested for common method bias using Harman's single-factor test. The two conditions are that there is more than one factor with eigenvalues greater than 1, and the maximum factor has a variance explained of less than 50% [35]. The results showed that there were 4 factors with characteristic root greater than 1, and the variance explanation of the first factor was 36.23%, which was less than the critical standard of 50%, indicating that there was no serious common method bias in the data of this study.

### 3.2. Descriptive Statistics

The median score of sleep quality was 6 (3, 10). Among them, 135 nurses (45.30%) reported good sleep quality (PSQI ≤5), while 163 nurses (54.70%) had poor sleep quality (PSQI >5), accounting for 54.70% of the total population.  Table 2 presents the demographic characteristics of psychiatric nurses. Their median age was 35 (31, 42.5), and the median length of service was 12 (8, 22).

### 3.3. Identifying the Potential Subtypes of Sleep Quality

The heterogeneity of sleep quality in psychiatric nurses is shown in Table 3. First, based on the scores of seven components of the PSQI scale, one to four latent profile models were sequentially fitted. When the number of profile models increased from 3 to 4, AIC, BIC, and aBIC values reached their minimum, while BLRT was still less than 0.001, and VLMR-LRT was greater than 0.05. However, the entropy value was lower than that of the 3-profiles model, indicating that the 4-profiles model was not better than the 3-profiles model. Finally, the 3-profiles model was chosen as the optimal model because its AIC, BIC, and aBIC values were close to the minimum, BLRT *P* value and VLMR- LRT *P* value were less than 0.05 (better than the 2-profiles model), it had the highest entropy value, and the class probability was more than 5%. The posterior probability of the latent profile of psychiatric nurses in the three-category model in this study was 100%, indicating that the model fit quality was high, and the model was reliable.

### 3.4. Latent Profile Characteristics According to the Profiles of Sleep Quality

**Figure 1:**
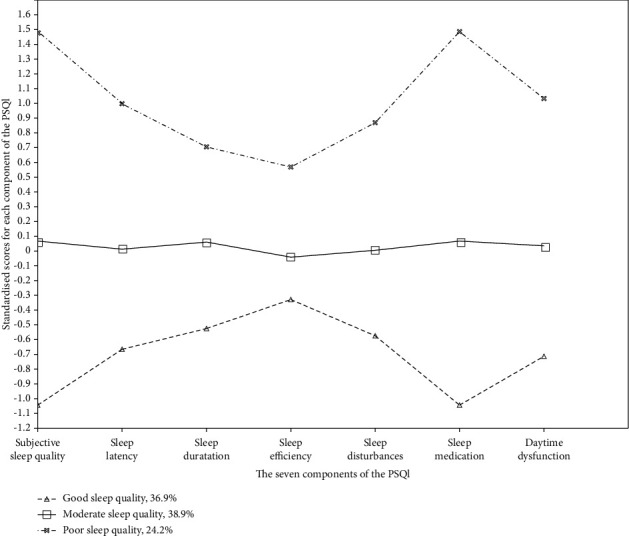
The result of latent profile analysis.

Table 4 and Figure 1 present the sleep quality scores for each profile identified in this study. Three distinct classes of nurses were identified. Class 1 comprised 110 nurses, accounting for 36.9% of the total population. This profile exhibited standardized scores lower than the other two profiles, with scores below zero in all seven components. Consequently, it was labelled as “good sleep quality”. Class 2 consisted of 116 nurses, representing 38.9% of the total sample. The standardized scores for various components of the PSQI indicated a moderate level of sleep quality, leading to its designation as “moderate sleep quality.” Class 3 included 72 nurses, making up 24.2% of the total participants. This profile demonstrated the highest standardized scores across all components, particularly in subjective sleep quality and sleep medication use. These findings suggest that the overall sleep quality of psychiatric nurses in class 3 is poor, with a higher frequency of sleep medication use and a general perception of poor sleep quality. Accordingly, this class was named “poor sleep quality.”

### 3.5. Kruskal–Wallis H Test of Factors Influencing Sleep Quality Profiles

As shown in Table 5, the Kruskal–Wallis *H* test indicated that there was a significant difference (*P* < 0.05) in age, marital status, education, work schedule, total weekly working hours, night shifts, special life events, nurse-patient relationships, and diseases among different potential profiles across three groups.

Post hoc comparisons using Dunn's method for multiple tests and the Bonferroni method were used to correct the *P* value to indicate that there were significant differences in age (corrected *P*=0.003), length of service (corrected *P*=0.007), marital status (corrected *P*=0.024), education (corrected *P*=0.025), working schedule (corrected *P*=0.001), total weekly working hours (corrected *P* < 0.001), night shifts (corrected *P* < 0.001), special life events (corrected *P* < 0.001), nurse-patient relationship (corrected *P*=0.002), and disease (corrected *P* < 0.001) between the good and poor sleep quality groups. There were significant differences in working schedule (corrected *P*=0.014) and total weekly working hours (corrected *P*=0.003) between the medium and poor sleep quality groups. There were significant differences in night shifts, special life events (corrected *P*=0.002) and disease (corrected *P*=0.009) between the good sleep quality group and the moderate sleep quality group.

### 3.6. Multivariate Logistic Regression Analysis of Factors Influencing Sleep Quality Profiles

Table 6 shows that nurses who are over forty years of age (OR = 2.600 and 95% CI: 1.186–5.698) and experienced special life events (OR = 2.966 and 95% CI: 1.355–6.495) were more likely to belong to the “moderate sleep quality” group compared to the “good sleep quality” group. Then, nurses who were over forty years of age (OR = 3.002 and 95% CI: 1.072–8.409) unmarried, divorced, separated, widowed (OR = 0.365 and 95% CI: 0.154–0.862), worked more than 40 hours per week (OR = 0.273 and 95% CI: 0.121–0.615), experienced specific life events (OR = 4.375 and 95% CI: 1.826–10.479), had a poor nurse-patient relationship (OR = 0.432 and 95% CI: 0.184–0.971), and had chronic diseases (OR = 2.910 and 95% CI: 1.354–6.252) were more likely to be in the “poor sleep quality” group compared to the “good sleep quality” group. In addition, nurses who worked 40 hours or less (OR = 0.442 and 95% CI: 0.212–0.920) were more likely to belong to the “moderate sleep quality” group compared to the “poor sleep quality” group.

## 4. Discussion

This study examined the heterogeneity of sleep quality among psychiatric nurses through different aspects of sleep quality. The study used an individual-centred approach to identify the subtypes of sleep quality among psychiatric nurses. The findings revealed that the main reasons for psychiatric nurses belonging to the “poor sleep quality” group were poor subjective sleep quality and frequent use of hypnotic medication. Moreover, the study investigated the impact of nurses' individual family life and work characteristics on their sleep quality, thereby opening up the perspective of the research on the influencing factors of sleep quality among psychiatric nurses.

### 4.1. Sleep Quality in Psychiatric Nurses

The median of PSQI score for the 298 psychiatric nurses in a mental health centre in Liaoning Province was 6 (3, 10). Among the participants, 135 nurses (45.30%) had good sleep quality (PSQI ≤5) while 163 nurses (54.70%) experienced poor sleep quality (PSQI >5). These findings indicate a relatively high incidence of poor sleep quality among psychiatric nurses in Liaoning Province, suggesting a more severe sleep problem that may be related to their professional characteristics and work pressure.

This study analyzed the sleep quality of psychiatric nurses using the LPA method. Based on the fitness indicators, the sleep quality of psychiatric nurses could be divided into the following three subgroups: the “good sleep quality” group, the “moderate sleep quality” group, and the “poor sleep quality” group.

The “moderate sleep quality” group had the highest number of nurses, which accounted for 38.9% of the total participants. The group exhibited relatively balanced scores across the seven components of the PSQI, indicating that more than one third of psychiatric nurses commonly experienced comprehensive sleep issues without any specific sleep problems. The “poor sleep quality” group of psychiatric nurses comprised 24.2% of the total participants, with the median PSQI score of 13 (11, 15). Within this group, subjective sleep quality and the use of hypnotic medication scored the highest compared to the other five components, suggesting that these two issues have a significant impact on impairing sleep quality. This may be attributed to the irregular behaviors and emotions displayed by psychiatric patients, which impose additional pressure and challenges on nursing work, leading to physical and mental burdens on nurses [10]. This contributes to poor sleep quality and increasing their demands for sleep medication.

### 4.2. Influencing Factors of Sleep Quality Profiles in Psychiatric Nurses

#### 4.2.1. Age

The findings indicated that nurses aged over 40 years were more likely to belong to the “moderate sleep quality” and “poor sleep quality” groups compared to the “good sleep quality” group. Chang et al. showed that nurses over 40 years old were more likely to have sleep disorders [36], and other studies proved that older age was a risk factor for poor sleep quality among nurses [37]. Most female nurses over 40 years old are experiencing menopause or menopause, and the fluctuation of body hormone levels affect their sleep quality [38]. In addition, older nurses may have to bear more responsibility and pressure at work and at home and are more likely to suffer from chronic diseases or other occupational diseases for a long time, which are the reasons for poor sleep quality of older nurses (especially those over 40 years old) [39]. Therefore, managers should provide enough rest time for older nurses to recover energy and balance various relationships, reduce the work intensity, arrange assistance personnel, and optimize work processes to improve the sleep problems of older nurses.

#### 4.2.2. Marital Status

The result indicated that unmarried/divorced/separated/widowed nurses were more likely to belong to the “poor sleep quality” group compared to the “good sleep quality” group, suggesting that these nurses had poorer sleep quality. Previous studies also had similar findings, showing that sleeping with the spouse can effectively improve sleep quality and overall health [40, 41]. Clinical and epidemiological studies consistently demonstrate that feelings of loneliness can have a detrimental impact on sleep quality [42]. Conversely, having a supportive spouse or partner has been found to contribute to better sleep quality. Moreover, the emotional support and sense of security provided by a significant other can positively influence an individual's mental wellbeing, further enhancing their sleep quality [43].

#### 4.2.3. Total Weekly Working Hours

The result indicated that nurses who worked more than 40 hours per week were more likely to belong to the “poor sleep quality” group compared to both the “moderate sleep quality” and “good sleep quality” groups. This finding aligns with previous studies that have shown a correlation between longer working hours and poorer sleep quality among nurses [44]. The reason may be that longer working hours need nurses to maintain a high level of concentration and endure long hours of standing to handle demanding nursing tasks. Both mental and physical fatigue can contribute to an increased sense of fatigue among nurses, thereby increasing their risk of experiencing poor sleep quality [45].

#### 4.2.4. Special Life Events

The findings of the study indicated that nurses who experienced special life events in the past year were more likely to belong to the “moderate sleep quality” and “poor sleep quality” groups compared to the “good sleep quality” group. Both negative and positive life events can have an impact on the sleep quality of psychiatric nurses. Previous research has also arrived at similar conclusions, stating that psychological stress resulting from negative events can affect the sleep quality of nurses [46]. However, there exists a complex relationship between positive life events, such as getting married, being promoted, or receiving awards, and sleep quality. Despite these events being positive, they can still lead to sleep disturbances due to arousal [47].

#### 4.2.5. Nurse-Patient Relationship

The study found that nurses who had a poor nurse-patient relationship were more likely to belong to the “poor sleep quality” group compared to the “good sleep quality” group. A discordant nurse-patient relationship has been identified as a significant predictor of increased work-family conflict [48] and has been found to have a negative correlation with job satisfaction [49]. The increase in work-family conflict and the decrease in job satisfaction can contribute to deterioration in sleep quality [50, 51].

#### 4.2.6. Disease

The result showed that nurses who had the disease were more likely to belong to the “poor sleep quality” group compared with the “poor sleep quality” group. Previous studies have also found a strong association between chronic disease and sleep quality [52, 53]. Nurses with chronic diseases are more likely to experience pain, fatigue, and negative emotions such as depressive symptoms, which can impair sleep quality [54, 55]. Chronic illness can also lead to changes in the social status, career, and family relationships, even increasing their financial burden. Dealing with and adapting to these changes can be psychologically stressful for nurses, which in turn increases the risk of poor sleep quality.

Nurses who were over forty years of age, unmarried/divorced/separated/widowed, working more than 40 hours per week, have experienced significant life events in the past month, had poor nurse-patient relationships, and had chronic diseases were more likely to have poorer sleep quality. In order to improve the sleep quality of older nurses, nursing managers should regularly communicate with nursing staff, assign work assistants, and help nursing staff establish a work-family balance. For nurses without partners, managers should provide adequate care and support. In addition, managers should develop flexible work schedules to help nurses balance clinical and research duties and avoid excessive work hours and fatigue. Managers should pay attention to the psychological state of nurses, understand the recent life experience of nurses, and communicate and intervene in time. Managers should also organize regular training sessions to enable nursing staff to build positive relationships with patients. The organization should organize regular physical examination, grasp the health status of nursing staff in time, arrange work reasonably for nurses with chronic diseases, and give them enough time for rest. In addition, psychiatric nurses who have poor subjective sleep quality and rely on the use of sleep drugs can improve their sleep quality through aromatherapy [56], adequate dietary nutrition [57], cognitive behavioral therapy [58], light therapy [59], breathing relaxation training [60], sleep education [61], exercise [62], and other intervention methods.

#### 4.2.7. Limitations

This study considered the heterogeneity of sleep quality among psychiatric nurses by examining different components and adopting an individual-centred perspective to identify variations within this group. The aim was to provide theoretical guidance and empirical evidence for targeted intervention strategies to improve their sleep quality. However, several limitations should be mentioned. First, this study is cross-sectional, which means that it could not establish exact causal relationships between variables. Second, the use of self-reported questionnaires introduces the potential for information bias in the study results. Third, the participants were limited to nurses from a specific psychiatric health centre in Liaoning Province, which may limit the generalizability of our findings. Fourth, this study only investigated whether nurses suffered from chronic diseases, ignoring that different types of chronic diseases may lead to different results. Therefore, future research should consider expanding the sampling scope and increasing the sample size and consider the impact of different kinds of chronic diseases on sleep quality of nurses.

## 5. Conclusions

This study used a person-centred approach and categorized the sleep quality of psychiatric nurses into the following three subgroups: good sleep quality, moderate sleep quality, and poor sleep quality. This suggests that for nurses with poor sleep quality, managers should prioritize addressing their subjective sleep quality and sleep medication use. Age, marital status, work schedule, total weekly working hours, night shifts, special life events, nurse-patient relationships, and chronic diseases were associated with their sleep quality. Managers need to pay attention to psychiatric nurses' sleep and take targeted intervention methods to improve their sleep problems. Individuals should also cultivate healthy behaviors and lifestyles to improve sleep quality.

## Figures and Tables

**Table 1:** The model fit indices for LPA.

Indicators	Criteria
AIC	The smaller the value, the more accurate the classification of the model
BIC	The smaller the value, the more accurate the classification of the model
ABIC	The smaller the value, the more accurate the classification of the model
Entropy	Entropy >0.8 means 90% of individuals are accurately classified
BLRT	The *P* value of <0.05 indicates that the class *k* model is superior to the class *k*-1 model
VLMR- LRT	The *P* value of <0.05 indicates that the class *k* model is superior to the class *k*-1 model
Probability of class	Models with a probability of at least 5% for each category were classified more reasonably

AIC, Akaike information criteria; BIC, Bayesian information criteria; aBIC, adjusted Bayesian information criteria; BLRT, bootstrap likelihood ratio test; VLMR-LRT, Vuong–Lo–Mendell–Rubin likelihood ratio test.

**Table 2:** Demographic characteristics of psychiatric nurses.

Variables	*n* (%)
Age (years)	
≤40	215 (72.1%)
>40	83 (27.9%)
Length of service (years)	
≤10	125 (41.9%)
>10	173 (58.1%)
Gender	
Male	101 (33.9%)
Female	197 (66.1%)
Marital status	
Married or cohabiting	216 (72.5%)
Unmarried/divorced/separated/widowed	82 (27.5%)
Education	
Junior college and below	82 (27.5%)
Bachelor's degree and above	216 (72.5%)
Monthly income (CNY)	
≤5000 yuan	112 (37.6%)
>5000 yuan	186 (62.4%)
Work schedule	
Fixed schedule	174 (58.4%)
Shift work	124 (41.6%)
Total weekly working hours (h)	
≤40	173 (58.1%)
>40	125 (41.9%)
Night shifts	
Yes	106 (35.6%)
No	192 (64.4%)
Special life events	
Yes	81 (27.2%)
No	217 (72.8%)
Nurse-patient relationship	
Good	232 (77.9%)
Poor	66 (22.1%)
Chronic diseases	
Yes	123 (41.3%)
No	175 (58.7%)

CNY is the standard currency symbol for China Yuan.

**Table 3:** Fitness indicators for potential profile models of sleep quality in psychiatric nurses.

Model	AIC	BIC	aBIC	VLMR- LRT (*P*)	BLRT (*P*)	Entropy	Probability of class
1	5940.800	5992.559	5948.160	—	—	—	—
2	5129.045	5210.381	5140.611	0.0037	<0.001	0.909	0.681/0.319
3	4384.762	4495.674	4400.533	0.0314	<0.001	1.000	0.369/0.389/0.242
4	4354.604	4495.094	4374.582	0.1411	<0.001	0.993	0.369/0.242/0.362/0.027

*Note.* LMR and BLRT provide *P* values as a measure of model significance. Abbreviation AIC, Akaike information criteria; BIC, Bayesian information criteria; aBIC, adjusted Bayesian information criteria; BLRT, bootstrap likelihood ratio test; VLMR-LRT, Vuong–Lo–Mendell–Rubin likelihood ratio test.

**Table 4:** Each PSQI component based on the profiles (M (P_25,_ P_75_)).

PSQI components	Good sleep quality	Moderate sleep quality	Poor sleep quality
Subjective sleep quality	0 (0, 0)	1 (1, 1)	2 (2, 3)
Sleep latency	1 (0, 1)	1 (1, 1)	2 (1, 3)
Sleep duration	0.5 (0, 1)	1 (0, 2)	2 (1, 2)
Sleep efficiency	0 (0, 0)	0 (0, 0)	0 (0, 1)
Sleep disturbances	1 (0, 1)	1 (1, 1)	2 (1, 2)
Sleep medication	0 (0, 0)	1 (1, 1)	2 (2, 3)
Daytime dysfunction	0 (0, 1)	1 (1, 2)	3 (2, 3)
PSQI	2 (1, 3)	7 (5, 9)	13 (11, 15)

*M* (*P*_25,_ *P*_75_): Median and interquartile range, PSQI: Pittsburgh Sleep Quality Index.

**Table 5:** Kruskal–Wallis *H* test of factors influencing sleep quality profiles.

Variable	Good sleep quality	Moderate sleep quality	Poor sleep quality	*H*	*P*
Age (years)					
≤40	65 (59.1%)^a^	89 (76.7%)^ab^	61 (84.7%)^b^	11.416	0.003
>40	45 (40.9%)	27 (23.3%)	11 (15.3%)		
Length of service (years)					
≤10	38 (34.5%)^a^	48 (41.4%)^ab^	39 (54.2%)^b^	9.952	0.007
>10	72 (65.5%)	68 (58.6%)	33 (45.8%)		
Gender					
Male	40 (36.4%)	41 (40.6%)	20 (27.8%)	1.605	0.448
Female	70 (63.6%)	75 (59.4%)	52 (72.2%)		
Marital status					
Married or cohabiting	87 (79.1%)^a^	85 (73.3%)^ab^	44 (61.1%)^b^	7.089	0.029
Unmarried/divorced/separated/widowed	23 (20.9%)	31 (26.7%)	28 (38.9%)		
Education					
Junior college and below	38 (34.5%)^a^	32 (27.6%)^ab^	12 (16.7%)^b^	6.951	0.031
Bachelor's degree and above	72 (65.5%)	84 (72.4%)	60 (83.3%)		
Monthly income (CNY)					
≤5000 yuan	36 (32.7%)	42 (36.2%)	34 (47.2%)	4.037	0.133
>5000 yuan	74 (67.3%)	74 (63.8%)	38 (52.8%)		
Work schedule					
Fixed schedule	74 (67.3%)^a^	71 (61.2%)^a^	29 (40.3%)^b^	13.627	0.001
Shift work	36 (32.7%)	45 (38.8%)	43 (59.7%)		
Total weekly working hours (h)					
≤40	79 (71.8%)^a^	69 (59.5%)^a^	25 (34.7%)^b^	24.668	<0.001
>40	31 (28.2%)	47 (40.5%)	47 (65.3%)		
Night shifts					
Yes	56 (50.9%)^a^	78 (67.2%)^b^	58 (80.6%)^b^	17.286	<0.001
No	54 (49.1%)	38 (32.8%)	14 (19.4%)		
Special life events					
Yes	12 (10.9%)^a^	36 (31.0%)^b^	33 (45.8%)^b^	28.146	<0.001
No	98 (89.1%)	80 (69.0%)	39 (54.2%)		
Nurse-patient relationship					
Good	95 (86.4%)^a^	90 (77.6%)^ab^	47 (65.3%)^b^	11.191	0.004
Poor	15 (13.6%)	26 (22.4%)	25 (34.7%)		
Disease					
Yes	28 (25.5%)^a^	52 (44.8%)^b^	43 (59.7%)^b^	21.997	<0.001
No	82 (74.5%)	64 (55.2%)	29 (40.3%)		

CNY is the standard currency symbol for China Yuan. *Note.* As long as there is the same marked letter, the difference is not significant. Any difference with different marked letters was considered significant (*P* < 0.05).

**Table 6:** Hierarchical multiple logistic regression analysis of factors influencing sleep quality profiles.

Variables	Reference: Good sleep quality			
	Moderate sleep quality		Poor sleep quality	
	OR (95% CI)	*P*	OR (95% CI)	*P*

*(a)*				

*Model 1*				
Age (reference: 40 years old or less) More than 40 years old	2.282 (1.285, 4.053)	0.005	3.839 (1.821, 8.096)	<0.001

*Model 2*				
Age (reference: 40 years old or less) More than 40 years old	2.353 (1.212, 4.562)	0.011	3.069 (1.288, 7.313)	0.011
Marital status (reference: married or cohabiting) Unmarried/divorced/separated/widowed	0.685 (0.303, 1.279)	0.261	0.317 (0.148, 0.677)	0.003
Education (reference: bachelor's degree and above) Junior college and below	1.050 (0.542, 2.063)	0.883	0.598 (0.256, 1.396)	0.235
Special life events (reference: no) Yes	3.016 (1.355, 6.495)	0.005	5.213 (2.248, 12.089)	<0.001
Disease (reference: no) Yes	2.002 (1.075, 3.728)	0.029	3.499 (1.675, 7.309)	0.001

*Model 3*				
Age (reference: 40 years old or less) More than 40 years old	2.600 (1.186, 5.698)	0.017	3.002 (1.072, 8.409)	0.036
Length of service (reference: 10 years or less) More than 10 years	0.866 (0.418, 1.793)	0.699	0.829 (0.342, 2.009)	0.677
Marital status (reference: married or cohabiting) Unmarried/divorced/separated/widowed	0.622 (0.303, 1.279)	0.197	0.365 (0.154, 0.862)	0.022
Education (reference: bachelor's degree and above) Junior college and below	1.058 (0.542, 2.063)	0.870	0.539 (0.221, 1.313)	0.174
Work schedule (reference: fixed schedule) Shift work	1.589 (0.735, 3.437)	0.239	2.829 (1.132, 0.453)	0.791
Total weekly working hours (reference: 40 hours or less) More than 40 hours	0.617 (0.312, 1.222)	0.166	0.273 (0.121, 0.615)	0.002
Night shifts (reference: no) Yes	1.291 (0.604, 2.758)	0.510	1.073 (0.408, 2.823)	0.887
Special life events (reference: no) Yes	2.966 (1.355, 6.495)	0.007	4.375 (1.826, 10.479)	0.001
Nurse-patient relationship (reference: good) Poor	0.707 (0.338, 1.480)	0.358	0.423 (0.184, 0.971)	0.042
Disease (reference: no) Yes	1.809 (0.954, 3.431)	0.069	2.910 (1.354, 6.252)	0.006

Variables	Reference: Moderate sleep quality			
	Poor sleep quality			
	*OR* (95% CI)		*P*	

*(b)*				

*Model 1*				
Age (reference: 40 years old or less) More than 40 years old	1.682 (0.777, 3.645)		0.187	

*Model 2*				
Age (reference: 40 years old or less) More than 40 years old	1.305 (0.561, 3.034)		0.536	
Marital status (reference: married or cohabiting) Unmarried/divorced/separated/widowed	0.463 (0.234, 0.916)		0.027	
Education (reference: bachelor's degree and above) Junior college and below	0.570 (0.257, 1.263)		0.166	
Special life events (reference: no) Yes	1.729 (0.884, 3.381)		0.110	
Disease (reference: no) Yes	1.714 (0.896, 3.406)		0.101	

*Model 3*				
Age (reference: 40 years old or less) More than 40 years old	1.155 (0.438, 3.045)		0.771	
Length of service (reference: 10 years or less) More than 10 years	0.957 (0.434, 2.109)		0.912	
Marital status (reference: married or cohabiting) Unmarried/divorced/separated/widowed	0.586 (0.272, 1.265)		0.173	
Education (reference: bachelor's degree and above) Junior college and below	0.510 (0.223, 1.166)		0.110	
Work schedule (reference: fixed schedule) Shift work	0.712 (0.313, 1.621)		0.419	
Total weekly working hours (reference: 40 hours or less) More than 40 hours	0.442 (0.212, 0.920)		0.029	
Night shifts (reference: no) Yes	0.831 (0.340, 2.031)		0.685	
Special life events (reference: no) Yes	1.475 (0.736, 2.953)		0.273	
Nurse-patient relationship (reference: good) Poor	0.598 (0.296, 1.208)		0.152	
Disease (reference: no) Yes	1.609 (0.812, 3.187)		0.173	

## Data Availability

The data that support the findings of this study are available from the corresponding author upon reasonable request. The data are not publicly available due to privacy or ethical restrictions.
